# Specific Activation of Estrogen Receptor Alpha and Beta Enhances Male Sexual Behavior and Neuroplasticity in Male Japanese Quail

**DOI:** 10.1371/journal.pone.0018627

**Published:** 2011-04-14

**Authors:** Aurore L. Seredynski, Gregory F. Ball, Jacques Balthazart, Thierry D. Charlier

**Affiliations:** 1 Research Group in Behavioral Neuroendocrinology, GIGA-Neurosciences, University of Liège, Liège, Belgium; 2 Department of Psychological and Brain Sciences, Johns Hopkins University, Baltimore, Maryland, United States of America; University of Akron, United States of America

## Abstract

Two subtypes of estrogen receptors (ER), ERα and ERβ, have been identified in humans and numerous vertebrates, including the Japanese quail. We investigated in this species the specific role(s) of each receptor in the activation of male sexual behavior and the underlying estrogen-dependent neural plasticity. Castrated male Japanese quail received empty (CX) or testosterone-filled (T) implants or were daily injected with the ER general agonist diethylstilbestrol (DES), the ERα-specific agonist PPT, the ERβ-specific agonist DPN or the vehicle, propylene glycol. Three days after receiving the first treatment, subjects were alternatively tested for appetitive (rhythmic cloacal sphincter movements, RCSM) and consummatory aspects (copulatory behavior) of male sexual behavior. 24 hours after the last behavioral testing, brains were collected and analyzed for aromatase expression and vasotocinergic innervation in the medial preoptic nucleus. The expression of RCSM was activated by T and to a lesser extent by DES and PPT but not by the ERβagonist DPN. In parallel, T fully restored the complete sequence of copulation, DES was partially active and the specific activation of ERα or ERβ only resulted in a very low frequency of mount attempts in few subjects. T increased the volume of the medial preoptic nucleus as measured by the dense cluster of aromatase-immunoreactive cells and the density of the vasotocinergic innervation within this nucleus. DES had only a weak action on vasotocinergic fibers and the two specific ER agonists did not affect these neural responses. Simultaneous activation of both receptors or treatments with higher doses may be required to fully activate sexual behavior and the associated neurochemical events.

## Introduction

Testosterone, the main steroid hormone produced by the gonads in male vertebrates, controls a wide range of physiological and behavioral responses, including male sexual behavior. In male quail (*Coturnix japonica*) like in other vertebrates, castration completely abolishes copulation while testosterone (T) treatment fully restores male sexual behavior [1], [2]. The behavioral action of T is largely mediated by its estrogenic and androgenic metabolites, 17β-estradiol (E_2_) and 5α-dihydrotestosterone (5α-DHT) respectively [3]–[5]. The conversion of T into E_2_ is catalyzed by the enzyme aromatase [6]–[8] which exhibits a very discrete distribution in the brain. A very dense expression of aromatase is notably found in the preoptic area and the hypothalamus [9]–[11].

The medial preoptic nucleus, POM is a sexually dimorphic (males >females) structure located in the medial preoptic area that plays a critical role in the control of male sexual behavior [12], [13]. While specific lesions of the POM completely abolish male sexual behavior, T implants stereotaxically positioned within the borders of the POM fully restore this behavior in castrated animals. In addition, numerous neurochemical markers of the POM are highly sensitive to circulating T concentrations. For example, aromatase-immunoreactive cells and vasotocin-immunoreactive fibers outline the entire POM and their density is significantly reduced in castrated birds but restored by exogenous T to a density that is typically seen in sexually mature males [14]–[17]. Interestingly, like male sexual behavior, the effects of T on aromatase and vasotocin expression are largely mediated by its estrogenic metabolites [18], [19].

Although numerous studies have also identified non-genomic or indirect genomic effects associated with the activation of membrane estrogen receptors [20], neural effects of estrogens are classically mediated via the activation of nuclear estrogen receptors (ER) [21]. The binding of E_2_ or of estrogen agonists to a nuclear ER induces its dimerization. The activated ER dimer then recognizes an estrogen response element (EREs) on the DNA and regulates the transcription of specific target genes [22]–[24].

There are two described nuclear estrogen receptor subtypes, α (ERα) and β (ERβ), and they have been identified in many vertebrate species including the Japanese quail [25]–[27]. Both subtypes have a discrete brain distribution that overlaps in numerous brain regions, including the POM and bed nucleus of the stria terminalis (BST) in quail [28], [29]. A similar distribution has been identified in mammals [30].

Kuiper and colleagues [31] hypothesized that estrogens differentially modulate reproductive and non-reproductive responses via two different ERs. Early work based on the specific distribution of the receptors and on specific ER knock-out mice (ERα ~KO and βERKO) suggested that ERα was mainly required for the normal development of reproductive organs, including uterus and gonads, and for expression of both male and female sexual behavior and while ERβ was responsible for the normal maintenance of non-reproductive organs such as the cardiovascular system or bones and for the regulation of social and cognitive aspects of the behavior (see for example:[32]–[35]). However, more recent functional analyses of the receptor actions tend to temper this simple dichotomy [36], [37]. Moreover, it was realized that the utilization of knockouts to study the distinct roles of ERα and β was limiting analysis to a single model species, did not discriminate between organizational and activational effects of the steroids and was potentially biased by compensatory mechanisms during development therefore rendering the generalization of ER function somewhat speculative. For example, the deletion of ERα differentially affects sexual behavior depending on the mouse backgrounds [38].

The development of specific receptor agonists allows a different approach to investigate the distinct functional role of estrogen receptor subtypes. Propyl-pyrazole-triol (PPT), an ERα specific ligand, and diarylpropionitrile (DPN), an ERβ specific ligand were developed thanks to the weak sequence identity of the C-terminal ligand-binding domain shared by two ER molecules, [39], [40]. DPN has an approximately 70-fold higher selectivity for ERβ over ERα while PPT is more or less 410 times more selective for ERα than ERβ [41], [42]. These compounds have been used to investigated the role of the two ERs in the control of various neuroanatomical, neurochemical and behavioral processes, including female sexual receptivity or proceptivity [43], aggressive behavior [44] or learning and memory [45]–[47] but to our knowledge, this approach has never been applied to the study of male sexual behavior.

The goal of the present study was to define the contribution of each estrogen receptor subtype in the estrogen-dependent activation of male sexual behavior and the underlying neuroplasticity in Japanese quail. Castrated male Japanese quail were daily injected with the general ER agonist diethylstilbestrol (DES) or one of the specific ER agonists and were tested for both appetitive and consummatory aspects of male sexual behavior. Both aspects of male-typical sexual behavior are under estrogenic control to a large extent though the site of estrogen action in activating each component of male behavior appears to be distinct to some degree [13], [48]. Negative and positive controls were provided by untreated castrates and by castrates treated with exogenous T. Two brain estrogen-sensitive responses, the volume of the POM defined by aromatase-immunoreactive staining and the density of vasotocin fibers in the POM were also investigated.

## Materials and Methods

### Animals and hormone treatments

Fifty-two male Japanese quail (*Coturnix japonica*) obtained from the breeding colony established in our laboratory were castrated at the age of three weeks as previously described [49]. Animals were housed in isolation and allowed to recover for at least 3 weeks. The subjects were then randomly distributed into six experimental groups: sixteen subjects were implanted subcutaneously in the neck region with two 20 mm-long Silastic™ tubes (Silclear ™ Tubing, Degania Silicone, 1.57 mm i.d., 2.41 mm o.d.) that were empty (CX group, n = 7) or filled with crystalline testosterone (Sigma, T group, n = 9). These testosterone-filled implants restore in castrated male quail physiological levels of the steroid that are typical of sexually mature males and produce a full activation of male sexual behavior [50]. The remaining birds were injected daily in the pectoral muscle for 13 days with the general ER agonist diethylstilbestrol (Sigma; 250 µg in 50 µl vehicle, DES group, n = 9), the ERα specific agonist 4,4′,4″-(4-Propyl-[1H]-pyrazole-1,3,5-triyl) trisphenol (Tocris, 250 µg in 50 µl vehicle, PPT group, n = 9), the ERβ specific agonist 2,3-bis(4-Hydroxyphenyl)-propionitrile (Tocris, 250 µg in 50 µl vehicle, DPN group, n = 9) or the vehicle propylene glycol (Sigma, 50 µl, PG group, n = 9).

The cloacal gland, an androgen-dependent structure [51], [52], was measured with callipers (greatest width × greatest length in mm^2^) before and at the end of the experiment to confirm the effectiveness of the treatments. The body mass was also recorded at the same times.

Throughout their life, birds were exposed to a photoperiod simulating long days (16 h light and 8 h dark per day) and had food and tap water available *ad libitum*. All experimental procedures were in agreement with the Belgian laws on the “Protection and Welfare of Animals” and on the “Protection of experimental animals” and were approved by the Ethics Committee for the Use of Animals at the University of Liège.

### Behavioral testing and brain collection

The subjects were tested daily, alternatively for copulatory behavior (consummatory sexual behavior) or for rhythmic cloacal sphincter movements (appetitive sexual behavior) starting three days after the beginning of the treatments. The behavioral tests started approximately 2 h after the daily injection. To assess consummatory sexual behavior, the experimental bird was introduced into a test arena (60×40×50 cm) that contained a sexually mature female with which the male could freely interact. During these tests, the frequency of sexual behavior patterns including neck grabs (NG), mount attempts (MA), mounts (M) and cloacal contact movements (CCM) (see [53], [54] for a detailed description of these behaviors) was recorded by an observer blind to the treatment of the birds. Six tests were carried out during the thirteen experimentation days.

To assess appetitive sexual behavior, the frequency of the rhythmic cloacal sphincter movements (RCSM) was quantified in a glass aquarium (40×20×25 cm) adjacent to another similar aquarium containing a female [55], [56]. A piece of opaque cardboard was attached to the exterior of the glass wall facing the experimenter to prevent the subject from being distracted by the presence of the experimenter. A mirror was placed at a 45° angle under the cage to allow the experimenter to view the cloacal area of the subject. A vertically sliding opaque panel was initially inserted between the two aquaria so that the experimental male could not see the female located in the second aquarium during 2.5 min. After this time, the sliding opaque panel was raised during the next 2.5 min. The male had then visual access to the female although he could not physically interact with her. The number of RCSM was recorded separately during the two 2.5 min periods when the male had or had not visual access to the female. The very low basal RCSM frequency observed in the absence of the female (usually less than 20) was then subtracted from the RCSM frequency observed in her presence to obtain a measure of the female-induced RCSM that is presented in the results. Four tests were carried out during the thirteen experimentation days.

Twenty-four hours after the last behavioral test and the last injection, birds were killed by decapitation and checked for the completeness of castration and presence of Silastic™ implants (when relevant). All birds were found to exhibit complete castrations and all subjects in the CX and CX+T groups still possessed their hormone implants. Brains were dissected from the skull, fixed in 5% acroleine in phosphate buffered saline (PBS, 90 min), rinsed twice in buffer and cryoprotected in 30% sucrose for 48 h. They were then rapidly frozen on dry ice and kept at −80°C until used.

### Immunohistochemistry

The brains were cut with a cryostat in the coronal plane from the level of the tractus septopallio-mesencephalicus to the caudal end of the tuberal hypothalamus. 30 µm-thick free-floating sections were collected in four series. Two series were stained by immunohistochemistry respectively for aromatase (ARO) and vasotocin (VT) as previously described and validated for quail (ARO: [11], [57]; VT: [16], [58]) with slight modifications. Briefly, sections were incubated for 15 min in 0.1% sodium borohydride in PBS and washed in PBS (pH 7.3–7.4). Endogenous peroxidase activity was blocked by incubating the sections for 20 min in 0.6% hydrogen peroxide, the non-specific antibody binding sites were blocked with 5% goat normal serum, and the sections were incubated with the primary antibody at 4°C (rabbit anti-quail recombinant ARO antibody, QR2/05 (gift from Prof. N. Harada, Fujita Health University, Toyoake, Japan) 1∶3000, overnight incubation) or rabbit anti-VT antibody (gift from Dr. D.G. Gray, Max Plank Institute of Bad Nauheim, Germany) 1∶5000, 48 hours incubation). Sections were then left for 2 h in secondary biotinylated goat anti-rabbit antibody (1∶400, Dako A/S, Gosltrup, Denmark) and finally incubated in ABC Vectastain elite Kit PK-6100 (Vector Laboratories) for 90 min. All reagents were in Phosphate-buffered saline 0.05 M containing 0.1% triton X-100 (PBST) and several rinses were performed between each step. The peroxidase enzymatic activity was then visualized with 3, 3′ diaminobenzidine tetrahydrochloride (DAB, 2%), 0.012% hydrogen peroxide in PBS (aromatase, ARO) or with DAB (2%), Nickel sulphate (25 mg/ml), 0.012% hydrogen peroxide in Sodium Acetate (0.175 M) (vasotocin, VT). Reaction was terminated by several rinses in PBS and the sections were mounted in an aqueous gelatin medium (aromatase) or in Eukitt® quick-hardening mounting medium (Sigma, vasotocin) and coverslipped.

### Image Analysis

**Figure 1:**
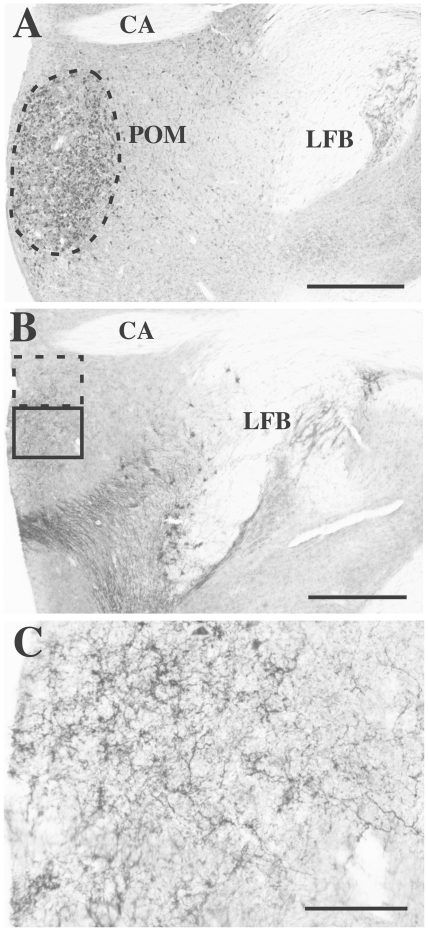
Photomicrographs illustrating the aromatase-immunoreactive perikarya (A) and the vasotocin-immunoreactive fibers (B, C) present within the medial preoptic nucleus (POM) that were quantified in the present study. Panel A illustrates the dense group of aromatase-immunoreactive neurons that outline the entire POM. The dotted line marks the limits of the POM as they were defined for quantification. Panel B shows the accumulation of vasotocin-immunoreactive fibers in the POM at the level of the anterior commissure. The rectangle drawn with a solid line indicates the area where quantification was performed that is illustrated at higher magnification in panel C. The dotted rectangle indicates how the camera field was originally placed before being moved to its final location (see text). Note that quantification of fibers concerned the steroid-sensitive network located in the POM, not the denser network located more ventrally that originates from the magnocellular neurons. CA: commissural anterior, LFB: latera forebrain bundle. Magnification bar =  500 µm in A–B, 100 µm in C.

Image acquisitions were performed by an observer blind to the treatment groups using a CCD camera (Model CFW-1612C, Scion Corporation, MD, USA) attached to an Olympus microscope and connected to a MacIntosh computer (Software: ImageJ, Wayne Rasband, NIH, Bethesda, MD, USA). We first calculated the volume of the medial preoptic nucleus (POM), defined by aromatase-immunoreactive-cells (See figure 1A.). The area of the nucleus was measured in all sections containing the nucleus throughout its rostro-caudal extent (objective 10X). Areas were then summed and multiplied by the sampling interval (120 µm; every 4^th^ 30 µm section was stained) to derive an estimate of volume. In addition, we analyzed the vasotocinergic innervation of the POM by calculating the relative optical density of the immunoreactive signal observed a with a 20 × objective. The quantification field (460×0.350 µm = 0.161 mm^2^ at 20X) was placed in the corner formed by the ventral edge of the anterior commissure and the lateral edge of the third ventricle at the level where the anterior commissure reaches its largest extension. The field was then moved one field ventrally (350 µm) and the optical density of the entire computer field at this location was quantified (see figure 1B–C). The relative optical density (ROD) was defined as the difference between of the optical density (gray levels) measured (after calibration) within the POM and in an equivalent area located in a vasotocin-free location in the telencephalon on the same section (background).

### Statistical analyses

Preliminary analyses indicated that there was, as expected, no significant difference between the two control groups (CX and PG) for all variables considered. Results from these two groups were thus pooled (CX/PG) in all statistical analyses presented here to increase their power.

The effects of treatments on the proportion of subjects displaying a specific behavior at least once during the six copulatory tests (NG, MA, M, CCM) or one the four RCSM tests was assessed by Chi square tests. One-tailed Fisher's exact probability tests were then used to identify a potential effect of specific compounds compared to the control group since increases only could possibly be observed (these behaviors are absent in castrated birds). The mean cumulative behavioral frequencies summed over all tests for each subject (active or inactive) were analyzed by non-parametric Kruskall-Wallis analyses of variance that were followed when appropriate by the post-hoc comparison of all experimental groups with the control group with the Dunn's test. All statistical results for NG and MA, on the one hand, and for M and CCM, on another hand, were nearly identical and to avoid redundancy we shall only present results relative to MA and CCM.

The size of the cloacal gland, the volume of the POM defined by aromatase-staining, and the density of vasotocinergic innervation in the POM were analyzed by parametric one-way analyses of variance (ANOVA) followed when appropriate by post-hoc Dunnett' tests comparing all groups with the controls.

All statistical analyzes were performed with GraphPad Prism 5.0 for MacOS X (GraphPad Software Inc, La Jolla CA) and all data are expressed as mean ± SEM. Differences were considered significant for p<0.05.

## Results

### Cloacal gland

**Figure 2:**
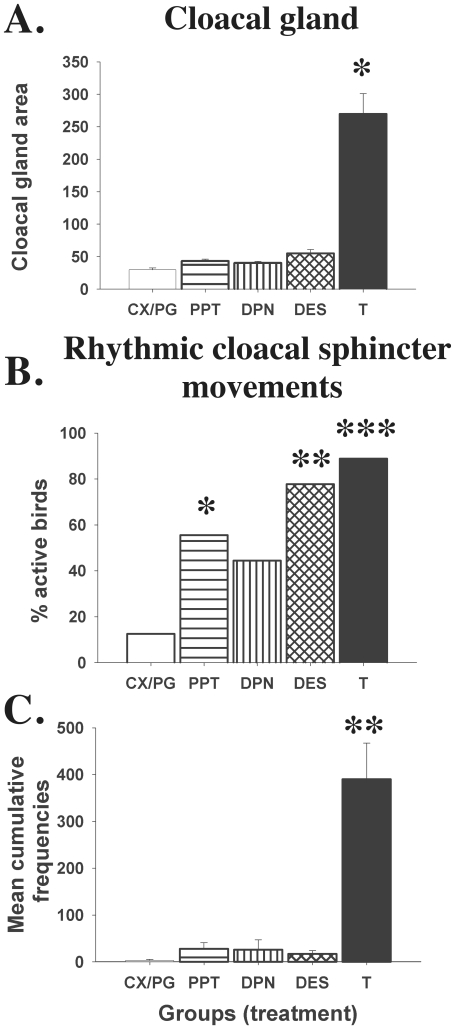
Cloacal gland area (in mm^2^) at the end of the experiment (A), percentage of birds that displayed at least one female-induced rhythmic cloacal sphincter movement (RCSM)(B) and cumulative frequencies of these RCSM in the 5 experimental groups (C). Data were analyzed by appropriate analyses of variance or c2 tests that were followed by post-hoc tests specifically comparing the 4 experimental groups to the controls (see text). Results of these post-hoc comparisons are shown at the top of the bars as follows: * =  p<0.05, ** =  p<0.01 and *** =  p<0.001.

Steroid treatments significantly affected the size of the cloacal gland (F_(4,47)_ = 59.71; p<0.0001, Figure 2A). Post-hoc comparisons by the Dunnett's multiple comparison test revealed a significant increase of the cloacal gland area in testosterone-treated subjects compared to CX/PG control group (p<0.01) while DPN, PPT and DES groups were not different from this control group (p>0.05).

### Appetitive Sexual Behavior

Treatments significantly influenced the proportion of birds displaying rhythmic cloacal sphincter movements (χ2 = 17.44, df = 4, p<0.0016). Two control birds showed RCSM very infrequently and only during one test while a significant number of birds treated with T (8/9), DES (7/9), PPT (5/9) or DPN (4/9) displayed this behavior at least during one test even if at low frequencies in some cases. These numbers of active birds were significantly higher than in controls in the T (p = 0.0003), DES (p = 0.0022) and PPT (p = 0.0343) groups but not following injection of DPN (p = 0.0972; all one-tailed Fisher's exact probability tests; see Figure 2B).

In addition, there was a general effect of the treatment on the RCSM frequency (H = 23.52, df = 4, p<0.0001; see Figure 2C). Specific post-hoc comparisons with the control group by the Dunn's test only revealed significant differences between the T and CX/PG groups (p<0.001).

### Consummatory Sexual Behavior

**Figure 3:**
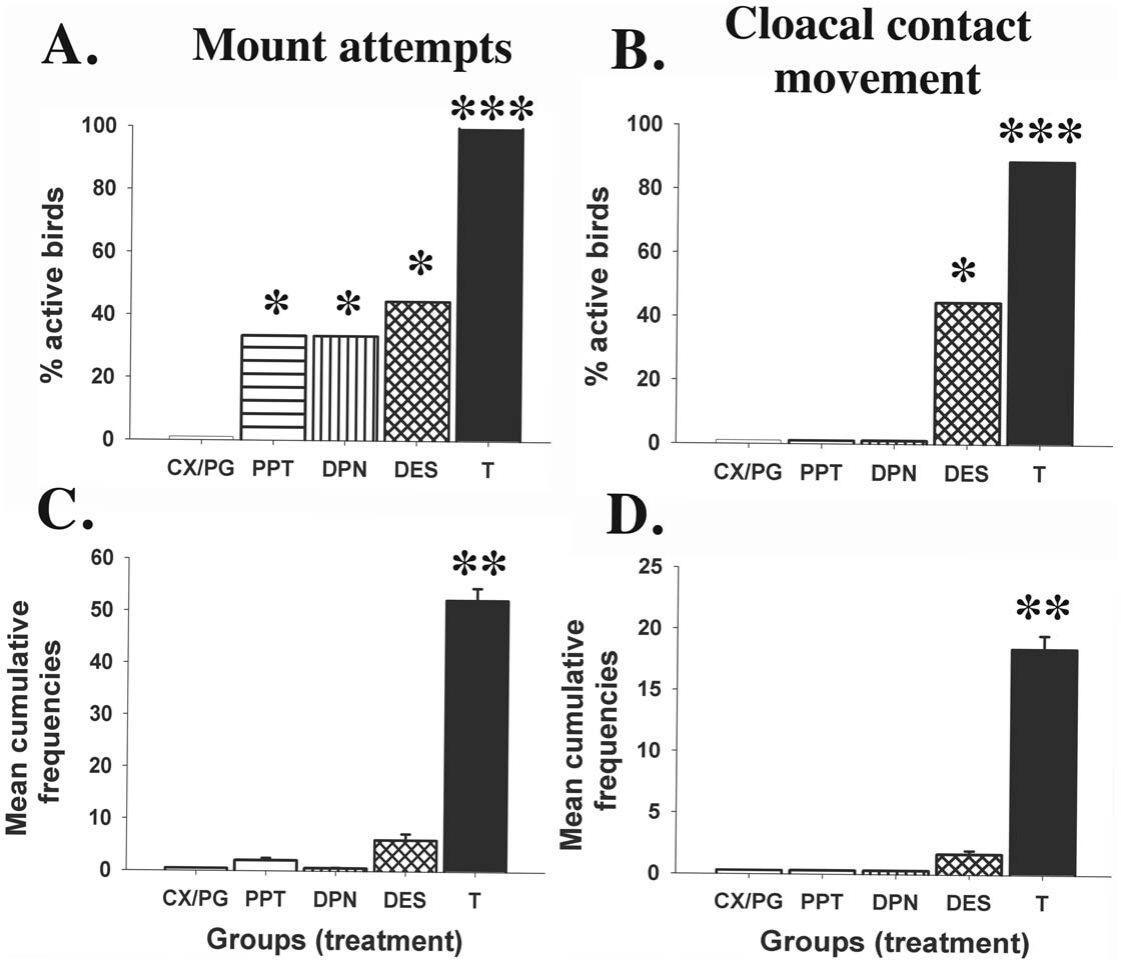
Percentage of birds that displayed at least one mount attempt (A) or one cloacal contact movement (B) and cumulative frequencies of these two behaviors in the 5 experimental groups (C–D). Data were analyzed by appropriate analyses of variance or χ2 tests that were followed by post-hoc tests specifically comparing the 4 experimental groups to the controls (see text). Results of these post-hoc comparisons are shown at the top of the bars as follows: * =  p<0.05, ** =  p<0.01 and *** =  p<0.001.

The endocrine treatments affected significantly the proportion of birds displaying MA (χ2 = 25.17, df = 4, p<0.0001) and CCM (χ2 = 34.47, df = 4, p<0.0001; Fig. 3A–B). As previously demonstrated, CX/PG subjects never showed any aspect of consummatory male sexual behavior while most T-treated birds displayed the full copulatory sequence (MA: 9/9, CCM: 8/9, p<0.0001 *vs.* controls). Approximately half of the DES birds were active (MA: 4/9, CCM: 4/9) which was also significantly different from controls (p = 0.01). Three out of the 9 birds treated with PPT or DPN showed MA (p = 0.0365) but never reached the end of the copulatory sequence (no M and no CCM) and were therefore not different from controls in this respect.

There was an overall significant effect of the treatments on the mean cumulative frequencies of consummatory sexual behaviors (MA: H = 33.32, df = 4, p<0.0001; CCM: H = 36.00, df = 4, p<0.0001). Post-hoc comparisons with the control group revealed significant differences between the T and CX/PG control group (MA and CCM: p<0.001) while mean cumulative frequencies of these behavior patterns were not different from controls in the other groups exposed to estrogenic stimulation (p>0.05 in all cases; see results for MA and CCM in Figure 3C–D).

### Aromatase immunoreactivity

**Figure 4:**
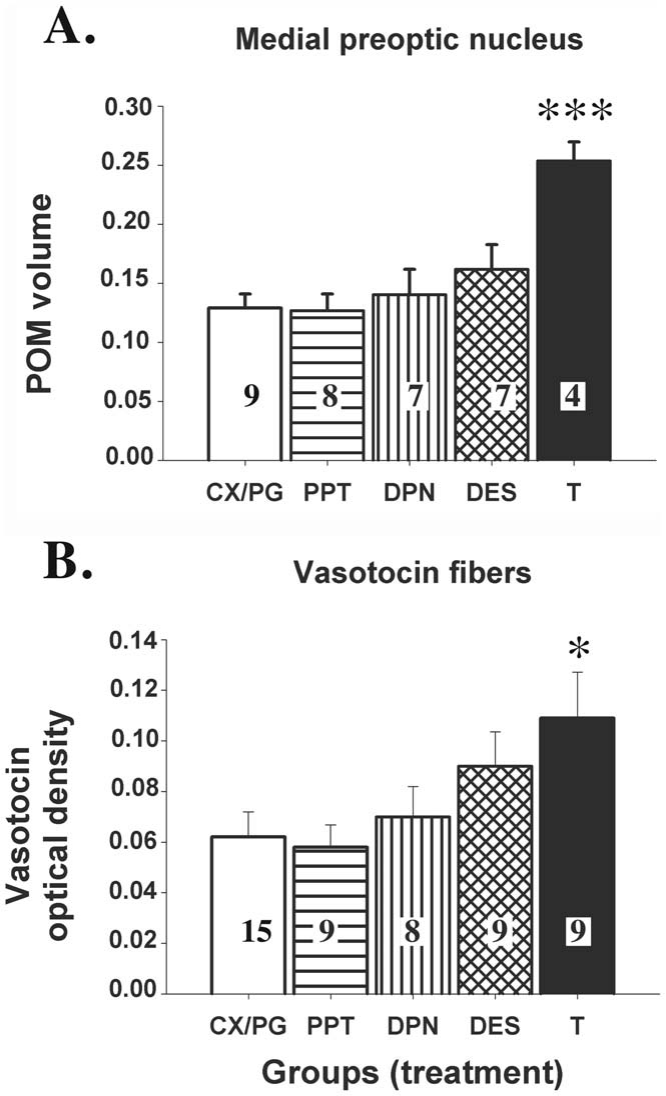
Volume of the medial preoptic nucleus (POM) as defined by the dense group of aromatase-immunoreactive neurons (A) and relative optical density of the vasotocin-immunoreactive fibers in the POM of the 5 experimental groups (B). Data were analyzed by one-way analyses of variance followed by post-hoc Dunnett's tests comparing the 4 experimental groups to the controls. Results of these post-hoc comparisons are shown at the top of the bars as follows: * =  p<0.05 and *** =  p<0.001. Since a number of brain samples were lost due to technical problems, the number of data points available for each group is shown on the corresponding bar in the graph.

Overall, the endocrine manipulations significantly affected the volume of the POM defined by the dense cluster of aromatase-immunoreactive cells (F_(4,30)_ = 10.80; p<0.0001). Post-hoc comparisons with the control group revealed that a significant increase in POM volume was present in the T group as compared to the CX/PG group (p<0.0001; see Figure 4A). The volume of the POM was, however, not different from the control group (p>0.05) in birds treated with DES, PPT or DPN.

### Vasotocin immunoreactivity

The vasotocinergic innervation of the POM, another steroid sensitive parameter, was quantified by measuring the relative optical density of the vasotocin-immunoreactive fibers in a standardized region of the nucleus observed at high magnification. An overall significant effect of the endocrine treatments was detected in this analysis (F_(4,45)_ = 4.068; p = 0.0067). Post-hoc comparisons revealed a significant increase in vasotocinergic innervation in T-treated birds as compared to the control group (p<0.01 see Figure 4B). The relative optical density of the vasotocinergic innervation was numerically higher in the DES-treated group than in controls but the difference did not reach statistical significance. PPT and DPN did not affect this measure.

## Discussion

We demonstrate here that the independent activation of estrogen receptors alpha or beta significantly affects separate aspects of male sexual behavior. This is the first study using specific ER agonists PPT and DPN to investigate independently the role of ERα and ERβ in the control of male sexual behavior in adult Japanese quail and, to our knowledge, in any vertebrate. Activation of ERα increased the expression of appetitive aspects of male behavior while both ERα and ERβ agonists, separately, enhanced the performance of the initial behavior patterns (NG and MA) in the copulatory sequence. Interestingly, the vasotocinergic innervation of the medial preoptic nucleus, a well-defined estrogen-dependent response, was no significantly affected by the general estrogen receptor agonist DES although the relative optical density was numerically higher compared to the controls. The independent activation of each receptor by specific agonists did not result in any increase.

### Appetitive sexual behavior

Male sexual behavior can be divided into an appetitive and a consummatory component. The appetitive aspect consists of searching and approaching a female while the consummatory aspect is the copulation *sensus stricto* [59]–[61]. Both aspects are known to depend on the presence of the estrogenic metabolites of testosterone [62]–[64] but the specific implication of each estrogen receptor subtype has not been investigated.

In quail, appetitive sexual behavior has been quantified by the measure of two specific behaviors: the learned social proximity response [62], [64]–[66] and the rhythmic cloacal sphincter movements (RCSM; [48], [55], [67]). These sphincter muscle contractions are used by reproductively active males just before copulation. The rhythmic contractions produce a foam that is transferred to the female cloaca and enhances fertilization success [55]. Both the social proximity response and RCSM disappear in castrated birds and are restored by treatment with exogenous testosterone [48]. While the activation of the social proximity response by testosterone requires the aromatization of the steroid [62], [68], the steroid specificity of RCSM activation and its dependence on estrogenic metabolites of testosterone is not so clearly established. One study showed that injection of testosterone-treated birds with an aromatase inhibitor decreases the expression of RCSM but this effect was only observed after 2–3 weeks of treatment with the inhibitor [69]. It is therefore possible that the blockade of testosterone metabolism did not affect directly the expression of RCSM but simply blocked the copulatory behavior *sensu stricto*, leading to a decrease of the reinforcing stimulus value of the female (see [69] for discussion). It was also shown that conditioned RCSM expressed after exposure to an arbitrary conditioned stimulus previously paired with the view of a female are inhibited by treatments with an aromatase inhibitor [56] but the effects of estrogens on this response have never been tested to our knowledge.

We show here for the first time that a general estrogen receptor agonist, DES, significantly increases the number of castrated males displaying RCSM in response to the visual presentation of a female. However, the frequency of these cloacal contractions was extremely low in comparison with birds treated with testosterone suggesting that other aspects of testosterone action (binding of testosterone or other androgenic metabolites to the androgen receptor) are likely to be critical for the activation of this behavior. The effects of DES were largely mimicked by injections of the ERα agonist PPT (increase in percentage of active birds but very low frequency of contractions), strongly suggesting that the activation of ERα is required to trigger RCSM in response to the female. On the other hand, no significant effect of the ERβ agonist DPN was observed on this behavior suggesting that this receptor is possibly not (less) implicated in the activation of this form of appetitive behavior.

Interestingly, the cloacal gland area increased markedly in testosterone-treated birds but remained small in animals treated with general or specific agonists of the estrogen receptors and in controls. This confirms the strict androgen-dependence of the morphological development of this gland [51], [52] whereas the activation of its contractions would be, at least in part, an estrogen-dependent phenomenon presumably resulting from an action of the steroids in the central nervous system. One key site of this action is likely to be the medial preoptic nucleus based on the fact that its lesion significantly inhibits the production of RCSM [48]. Importantly, we know that this nucleus expresses very high levels of both estrogen receptors subtypes, making it a ideal target for estrogen actions on appetitive behaviors, including RCSM ([28], [29], [70], [71] see below).

### Consummatory sexual behavior

A large number of studies have established that consummatory aspects of male sexual behavior are activated in castrated male quail by both testosterone and estrogens [1], [3], [8]. Additionally, effects of testosterone on these behaviors are blocked by the concurrent administration of either aromatase inhibitors [8], [72] or anti-estrogens [73], [74] thus indicating that testosterone must be aromatized into estrogens to exert these behavioral effects. Accordingly, we show here that both testosterone and DES-treated birds displayed the full copulatory sequence. It should however be noted that behavior activation in the DES-injected group concerned a smaller percentage of subjects that displayed the behaviors with much lower frequencies than testosterone-treated birds, as was the case in preceding studies [74]. The two specific ER agonists activated NG and MA roughly to the same extent as DES (±40% of birds active but with low frequencies) but had absolutely no effect on the expression of CCM. This would suggest that both ER subtypes are playing a similar role in the activation of consummatory behavior but the low level and incomplete activation of copulatory sequence obtained here limits the significance of this conclusion (see also below).

### Central effects of specific ER agonists

#### POM volume as defined by aromatase

We also investigated the effect of PPT and DPN administration on the volume of the medial preoptic nucleus (POM) as defined by aromatase immunoreactivity. As mentioned in the introduction, the POM boundaries are outlined by the presence of a dense group of aromatase-immunoreactive neurons. Aromatase activity and the numbers of aromatase-immunoreactive cells in POM are enhanced by testosterone and this effect of testosterone is mediated by the synergistic action of its androgenic (5α-DHT) and estrogenic (E2) metabolites both in birds including quail [75], [76] and in mammals [77] with the most prominent role being played by estrogens in birds. The induction of aromatase expression in POM usually parallels and is thought to contribute markedly to the activation of male copulatory behavior [78], [79].

Somewhat surprisingly, we observed here only minimal effects of DES administration on the POM volume defined by aromatase-immunoreactive neurons and PPT or DPN had absolutely no effect on this measure despite the fact that testosterone-treated birds showed, as expected [12], [78] a doubling of POM volume. The exact mechanism of estrogen action on aromatase expression is unclear. Aromatase-immunoreactive neurons co-express androgen receptors [80] but their colocalization with estrogen receptors is less well defined in the POM. Both ERα and ERβ are densely expressed in the POM [28], [70], [71], [81]. However, cells expressing ERα do not exhibit a colocalization with aromatase in this region (they are located in adjacent but different cells [81], and the co-localization with ERβ has not been investigated to this date. These data therefore suggest that it is possible to activate weakly some aspects of male sexual behavior without increasing aromatase expression as measured by the volume occupied by imunoreactive perikarya. This weak induction would therefore depend on the activation of other cell types.

#### Vasotocinergic innervation of POM

As expected based on previous studies [18], the density of vasotocin-immunoreactive fibers was increased here following treatment with testosterone and to some extent with the general estrogen receptor agonist DES. Injections of specific ER agonists had however no effect on the density of vasotocinergic fibers. These fibers originate mostly in neurons located in the medial part of the BST [82]. The type(s) of estrogen receptors expressed by these neurons has not been investigated in quail although this nucleus presents an intense expression of both ERα and ERβ [28], [71], [81].

Similarly in mammals, the exact implication of a specific type of receptor involved in the control of vasopressin, the mammalian homologue of vasotocin, in the BST is currently not clear. The presence of a functional estrogen response element in the promoter region of vasopressin suggests a direct control of expression by estrogens and it was actually reported that both ERα and ERβ were able to enhance the transcription [83]. Vasopressin-synthesizing cells from the BST, as defined by the presence of neurophysin-immunoreactive material (vasopressin transporter) were shown to express ERα [84] but these receptors were apparently not present in the magnocellular vasopressin neurons of the supraoptic and paraventricular nuclei. In contrast, these magnocellular neurons express ERβ [85]–[87] but studies of ERβ in the BST have apparently not been carried out. It is interesting to note here that, although previous work indicates that the sexual differentiation of the vasotocinergic parvocellular system during development is controlled by estrogens [88] the injection of PPT in quail eggs had no effect on the density of these fibers in adult quail [89].

### Neuroanatomical distribution of ERα and ERβ in quail and the neural site of estrogen action

The distribution of estrogen binding sites was first described in quail by in vivo autoradiographic procedures that did not discriminate between the two receptors subtypes [90]. The development of immunohistochemistry and *in situ* hybridization, as well as the discovery of two estrogen receptor subtypes subsequently allowed a more precise localization and functional understanding of estrogen action. In the quail brain, the distribution of ERα and ERβ has been studied by *in situ* hybridization localizing brain areas that express the corresponding mRNA [28], [29], [71] and, for ERα only, by immunohistochemistry of the receptor protein [70], [81]. All these studies concur to indicate that both receptor subtypes are present in all nuclei that have been shown to be implicated in the control of male reproductive behavior by lesion and/or steroid implantation experiments. A similar situation has been observed in the other vertebrate, including rats and mice [30].

The nuclei expressing these receptors include the medial preoptic nucleus (POM) that has been implicated in the control of appetitive and consummatory aspects of male sexual behavior (see review in [13], [63]), the medial nucleus of the stria terminalis, whose lesion decreases expression of copulatory behavior [48], and the nucleus taeniae of the amygdala that seems implicated in the control of both appetitive and consummatory sexual behavior although conflicting results have been reported for this nucleus [67], [91].

Based on these neurochemical data, it is impossible to determine the anatomical sites where specific ERα and ERβ agonists act to activate appetitive and/or consummatory sexual behavior. The POM is to this date the only nucleus where direct effects of general estrogens agonists were shown to activate aspects of male sexual behavior [92]. However, the wide distribution of the two ERs and of aromatase, in parallel with the observation that testosterone acts at multiple levels in the brain to control behavior strongly suggests that other brains sites must be implicated and the precise localization of neural substrate affected by steroids deserve additional study.

### Behavioral effects of ERα and ERβ activation in other species

Since the discovery of a second ER, numerous studies have tried to delineate precisely the role of each receptor in the control of various estrogen-dependent physiological and behavioral responses. ERα and ERβ knock-out (KO) mouse models as well as double knock-outs (ERαβKO) have played an important role in this research and demonstrated an implication of one or both receptors in the control of male and female sexual behavior [93]–[97], male aggressive behavior [98]–[100], social interactions and anxiety [101], [102] and in the activity of various neurochemical systems [103], [104].

It was originally assumed that ERβ was mainly inhibiting ERα action [31] but the situation was later shown to be more complex. Multiple studies have now demonstrated that the two subtypes of ERs may act independently, synergistically or antagonistically to regulate brain function and behavior [37], [105]. One limitation of these studies is, however, that they cannot discriminate between organizational and activational effects of the steroids and that the suppression of the receptors from the earliest developmental stages might activate compensatory mechanisms. The transient activation of one of the two receptor subtypes with specific agonists avoids these problems these problems and has also been used in a number of behavioral and physiological studies in mammals.

Studies with these specific agonists have confirmed the existence of a dissociation of the functions of ERα and ERβ. For example, PPT (ERα agonist) but not DPN (ERβ agonist) elicits sexual proceptivity (ear wiggling, hopping and darting) and receptivity (lordosis) in the female rat [43], [106], [107]. Furthermore, the co-administration of DPN with PPT decreased the PPT-induced expression of both proceptive and receptive female sexual behavior, suggesting that ERβ could be a modulator of ERα-dependent expression on female sexual behavior [43]. Surprisingly, this approach has, to our knowledge, never been used to study the activation of estrogen-dependent aspects of adult male sexual behavior. It should however be noted that *in ovo* treatment with PPT demasculinized male copulatory behavior in Japanese quail [89].

### … and in quail?

The present results suggest that both ERα and ERβ are implicated in the control of brain and behavior in quail. The activation of these two receptors subtypes has similar effects on some responses (mount attempts) but different impacts on others (RCSM). The magnitude of these effects was however limited and additionally a number of responses that are known to be estrogen-dependent were not affected here by PPT or DPN. Several factors must be considered to interpret this partial specificity and small magnitude of effects.

First, the doses of PPT and DPN that were selected here for testing (250 µg/birds/day i.e., ±1.25 mg/kg/day considering an average body weight of 200 g) might have been insufficient to fully reveal the effects of the activation of ERα and ERβ. These doses were however in the range of doses used in previous studies demonstrating effects on various behavioral or neurochemical responses in mammals (e.g., [43], [44], [46], [47], [108]). In addition, they were selected in an attempt to preserve the selectivity of these two agonists. Even if PPT has approximately 350–400 times more affinity for the ERα than for ERβ (Ki relative to estradiol of respectively 0.50 and 700 nM) and conversely DPN has 70–80 times more affinity for ERβ than ERα (respective Ki equal to 2.5 and 195 nM) (see [35], [41], [42]) some cross-reactivity will occur *in vivo* if subjects are injected with higher doses.

It is also noteworthy that DES has a higher affinity for the two receptors (0.13 and 0.15 nM for ERα and ERβ respectively, similar to estradiol; [26] compared to the specific agonists for their respective receptors (PPT: Relative binding affinity for ERα where E_2_ is 100%: 20–50%; DPN: Relative binding affinity for ERβ where E_2_ is 100%: 5–20%; [41], [42], [109]. This lower affinity of the specific agonists for their receptor should contribute to explain the smaller amplitude of behavioral and physiological response compared to the general agonist DES. In addition, no information is to our knowledge available on the stability of the agonists in vivo.

The doses selected here ensured a reasonable discrimination of receptor activation at physiological concentrations. Because effects observed here had a small amplitude (namely compared to DES and T), one can thus be reasonably sure that effects were mediated by the activation of the receptor that was targeted. Future work should however investigate the effects of higher doses in order to ascertain whether the limited amplitude of effects observed here reflects the intrinsic properties of these compounds or relates to the doses that were used.

Alternatively, it is also possible that some of the responses investigated here may be stimulated following a simultaneous activation of both ERα and ERβ. This would explain why DES and estradiol itself activate a number of responses that were not affected here by the specific agonists. The study of female behavior in rats rather suggests that DPN inhibits PPT action on female sexual behavior [43] but synergistic effects might also exist for other responses.

### Conclusion

Altogether, these results suggest a key role of both receptors at different level in the control of male sexual behavior although they do not allow to fully discriminate between the specific roles of ERα and ERβ. The relatively weak activation of the behaviors observed here might be due to the use of insufficient doses of agonists, to a requirement for synergistic activation of both ERα and ERβ or to differences between avian and mammalian receptors. Future studies should address these questions by a combination of behavioral and biochemical approaches.
